# Co-creation of a gender responsive TB intervention in Nigeria: a researcher-led collaborative study

**DOI:** 10.1186/s12913-025-12241-7

**Published:** 2025-01-13

**Authors:** Chukwuebuka Ugwu, Oluwatoyosi Adekeye, Beate Ringwald, Rachael Thomson, Obioma Chijioke-Akaniro, Chukwuma Anyaike, S Bertel Squire, John Bimba, Tom Wingfield

**Affiliations:** 1 Liverpool School of Tropical Medicine, Liverpool, UK; 2 Zankli Research Centre, Bingham University, Karu, Nigeria; 3 Obioma Chijioke-Akaniro, National Tuberculosis Leprosy and Buruli-Ulcer Control Programme, Federal Ministry of Health, Abuja, Nigeria; 4 Karolinska Institutet, World Health Organization Collaborating Centre On Tuberculosis and Social Medicine, Department of Global Public Health, Stockholm, Sweden; 5 Tropical and Infectious Disease Unit, Liverpool University Hospitals NHS Foundation Trust, Liverpool, UK

**Keywords:** Active TB Surveillance, Participatory Research, Stakeholder Engagement, Gender-Response

## Abstract

**Background:**

In Nigeria, men constitute over half of the people notified with tuberculosis (TB), experience longer delays before reaching care, and are estimated to account for two thirds of people who miss out on care. The higher TB risk and burden in men has implications for the whole population and reaching them earlier with TB services will reduce onward transmission in households, communities, and workplaces. The absence of a comprehensive guidance and the lack of substantial empirical evidence on TB care approaches that are responsive to the needs of men in Nigeria exacerbates this problem. Therefore, this research aimed to co-create a gender-responsive intervention for men in peri-urban communities in Nigeria.

**Methods:**

Our study utilised a researcher-led collaborative approach to engage local TB stakeholders including communities adversely affected by the disease to co-create a gender-responsive TB intervention. Between March and November 2022, we engaged 13 local TB stakeholders in a three-phase participatory intervention design process. This engagement involved two iterative cycles of Delphi research online, and an in-person workshop. In the first and second phases, participants described the potential impact of 15 listed interventions and prioritised combinations of nine interventions deemed to be effective in overcoming identified gendered barriers. Responses were analysed using a combination of qualitative framework approach, content analysis, and summary descriptive statistics assisted by NVivo software. Stakeholder consensus on a preferred intervention package was reached during the participatory workshop.

**Results:**

Overall, participants prioritised approaches that sought to actively find and systematically screen men for TB including awareness creation as a crucial component. The stakeholders placed significant considerations on the synergy between interventions and their programmatic sustainability when making their final choices. Consequently, a complex intervention package comprising three components was developed. These included targeted awareness creation among men in communities; TB screening in male-dominated socio-cultural congregate settings; and the use of digital chest X-ray screening. Anticipated early outputs of this intervention included improved TB knowledge, increased care-seeking, reduced TB-related costs and TB stigma, and accelerated early diagnosis among men in Nigeria.

**Conclusion:**

Leveraging the insights and experiences of local stakeholders through iterative engagements yielded consensus on a viable gender-responsive TB intervention.

**Supplementary Information:**

The online version contains supplementary material available at 10.1186/s12913-025-12241-7.

## Background

Gender has emerged as an important factor shaping the global tuberculosis (TB) epidemic. According to the WHO 2022 global TB report, 3.8 million men (57%), 2.4 million women (36%), and approximately half a million children aged 0 to 14 years (7%) were diagnosed and notified with TB in 2021 [1]. This pattern, in which men constitute the majority of all registered and treated TB cases, has remained consistent within WHO global data over the past decades [2]. Further global evidence from WHO shows that the incidence of TB is higher among men and that men constitute the majority of people with TB who go undiagnosed, untreated, or unreported [3]. Likewise, a meta-analysis of nationwide TB surveys shows that, in sub-Saharan Africa, men bear a higher burden of TB and account for two-thirds of people with undiagnosed TB [4].

Nigeria, faced with a triple burden of TB, drug-resistant TB and HIV-associated TB [1] and a TB incidence rate of 219 per 100,000 people per year bears Africa’s highest TB burden and sixth highest globally [5]. The TB burden in Nigeria is highly gendered with TB prevalence among men being more than two-fold that of women (751 [95% CI: 538 – 965] vs 359 [95% CI: 213 – 505] cases per 100,000 population) [6]. An estimated 70% of people with TB disease missed out on care in Nigeria [3]. Although men constitute the majority (56%) of people receiving treatment for TB [3], many experience delays in reaching TB diagnosis and care. Challenges to accessing TB services are reflected by the prevalence-to-notification ratio of 7.2 for men and 4.6 for women [6], contributing to adverse clinical and socioeconomic outcomes as well as increasing the risk of TB exposure among their contacts. Data from the latest WHO Global TB report shows that adolescent boys and young men between the ages of 15–24 have the lowest treatment coverage of all people above 15 years with TB, regardless of gender, in Nigeria [2]. The vulnerabilities to acquire and develop TB are exacerbated in underserved, informal peri-urban settlements due to high population density, household overcrowding, and limited access to routine health services [7].

Previous qualitative research identified significant barriers to TB care among TB affected peri-urban communities in Nigeria. The findings revealed that men in these settings have poor access to information about TB and available services. This is compounded by male gender norms, roles, and societal expectations which create bottlenecks for men and hinder their timely access to available TB services [8, 9]. For most men in informal work situations seeking care usually implies a forfeiture of the day’s wage, which can directly impact their family’s food security and survival [10]. TB-related stigma coupled with dismissive attitudes – often unknowingly – towards early TB symptoms and unwillingness to engage with care in formal healthcare facilities deter men from accessing TB care. Health systems capacity to provide care and services in ways that are welcoming to men and respond to their needs and preferences is limited [9].

In recent years, the Nigerian TB programme has made notable improvements in reaching people with TB, although achievements in reducing TB notification gap are greater among women than men [11]. The political declarations of the 2018 UNHLM specifically called for gender-responsive TB services to improve TB treatment outcomes and prevent TB transmission by reaching men with active TB [12]. However, there is no clear or unifying guidance on gender-responsive TB services in Nigeria. Gender-responsive TB interventions must effectively address context-specific barriers faced by men. Whilst the involvement of in-country stakeholders is a key factor contributing to the success and impact of research and programmes [13], they were found to be largely absent from the prioritisation processes relating to TB [14]. This paper explains the consensus-building process our research team facilitated with key stakeholders in Nigeria, to identify a locally appropriate TB intervention designed to address gendered barriers faced by men within peri-urban communities in Nigeria. The identified intervention will be evaluated in a research setting.

## Methods

This formative research was conducted as part of the “Developing and Evaluating gender-responsive TB Interventions for communities in Nigeria” (DESTINE) study. The mixed methods implementation study DESTINE is affiliated with the Leaving no one behind; transforming gendered pathways to health for TB (LIGHT) Consortium [15]. LIGHT is a six-year cross-disciplinary global health research programme led by LSTM in collaboration with partners in Kenya, Malawi, Nigeria, Uganda, and the UK. LIGHT aims to support policy and practice in transforming gendered pathways to health for people with TB in urban settings.

### Study setting

Nigeria is Africa’s largest economy [16] and most populous country [17] and home to over 108 million males, reporting a male to female ratio above the global average (1.02 vs 1.01) [18]. Significant gender disparities exist in Nigeria with men having better education, more employment, and earn more money than women [19]. However, men utilise health services less than women and have significantly shorter life spans [20]. TB is the third highest killer of males in Nigeria, after neonatal conditions and lower respiratory tract infections. The estimated male TB mortality rate of 81 per 100,000 is nearly twice that of females [21]. The three levels of government, federal, state, and local government, loosely correspond to the tertiary, secondary, and primary levels of care in the country. TB diagnostic and treatment services are mostly donor-funded and rendered through TB clinics at all care levels, which constitute 47% of all existing formal healthcare facilities in the country designed and guided by the Federal Ministry of Health [1, 22].

### Design

We used a researcher-led collaborative study design [23] to facilitate a consensus building process with key stakeholders in Nigeria. This approach was selected for its proven potential to garner perspectives, facilitate stakeholder buy-in and collaboration, and generate interventions tailored to the social and health system context – in this case, a gender-responsive intervention for men with TB in Nigeria. Consensus building approaches have been used to gauge the level of consensus amongst experts and/or lay people in health systems research [24] for guideline development [25] and setting standards of care [26] amongst others [27–29]. Our consensus building process evolved through three phases that sought to (1) identify potential interventions, (2) group the interventions into synergistic packages, and (3) identify the most viable package. The first two phases utilised the Delphi research method [24] followed by a participatory stakeholder workshop (Table 1). It is important to note that experts’ consensus around potential strategies to address public health issues may not always prove effective in addressing the targeted challenges. However, this paper focuses on reporting our approach towards consensus building.

**Table 1:** Summary of the consensus building process

Research stage	Outcome	Method and activities	Participants
Literature review	To identify potential interventions to improve access to TB diagnosis and care among men in peri-urban settlements in Nigeria	Scoping review	Multi-disciplinary research team (*n* = 7)
Stakeholder engagement 1	To collect expert opinion on the potential of interventions to improve access to TB diagnosis and care among men in peri-urban settlements in Nigeria	-Delphi method (online) -Open ended questions (email) -Stakeholders described potential interventions -Clarifications were made after initial responses	Stakeholders (*n* = 13)
Data analysis 1	To identify most viable interventions for inclusion in a TB intervention package to improve access to TB diagnosis and care among men in peri-urban settlements in Nigeria	-Qualitative data analysis using Framework approach [30] -Indexing and summarising data using NVivo12 pro (Lumivero USA)	Multi-disciplinary research team (*n* = 7)
Stakeholder engagement 2	To collect expert opinion on potential packaging of identified TB interventions to improve access to TB diagnosis and care among men in peri-urban settlements in Nigeria	-Delphi method (online) -Structured questionnaire (online form) -Stakeholders packaged interventions likely to work synergistically when co-implemented	Stakeholders (*n* = 13)
Data analysis 2	To identify the most-frequently-packaged combination of interventions to improve access to TB diagnosis and care among men in peri-urban settlements in Nigeria	-Descriptive summary statistics -Content analysis [31] of the written justification provided for the packages	Multi-disciplinary research team (*n* = 7)
Stakeholder workshop	To discuss and reach consensus on the most viable TB intervention package for improving access to TB diagnosis and care among men in peri-urban settlements in Nigeria	-Participatory stakeholder workshop (in-person)	Stakeholders (*n* = 13)

The research team and stakeholders remained the same throughout the study, except two stakeholders, who agreed to take part but did not respond during stakeholder engagement 1 and did not participate in subsequent research activities

## Research participants

We used a key informant sampling approach to select individuals who had worked for at least ten years in TB coordinating systematic (active or passive) screening for TB disease in Nigeria. We also considered individuals successfully treated for TB and since then actively participated in TB advocacy. Given that a relatively small group of similarly trained and experienced persons can yield reliable data for consensus building [25], we considered that 12 to 16 participants would enable us to achieve the study objectives. We initially identified and invited 17 potential participants of whom 15 accepted and 13 started and completed the process. The lead researcher CU called selected participants on phone; explained study objectives, methods, and activities; and afterwards sent participants information sheet and consent form per email. All participants provided written informed consent prior to their involvement in the study. Here we present data from 13 TB stakeholders (7 men, 6 women) who participated in all phases of the study (Table 2).

**Table 2:** TB stakeholders engaged in the consensus building

Participants	Gender	Organization Type	Level of work	Area of specialty
P1	F	CBO	Local government/ community	Advocacy, communication, social mobilisation, systematic (active) screening for active TB
P2	M	Academia	State	TB implementation research
P3	F	CSO	State	Public–Private mix, systematic (active) screening for active TB, monitoring & evaluation
P4	M	NTP	State	Programme management, systematic (active) screening for active TB
P5	M	Technical partner	Regional	Systematic (active) screening for active TB, capacity building
P6	M	Technical Partner	Regional	Systematic (active & passive) screening for active TB
P7	M	Technical partner	Regional	Monitoring & evaluation
P8	F	CBO/CSO	National	Advocacy, communication, social mobilisation
P9	M	CBO/IP	National	Advocacy, systematic (active) screening for active TB
P10	M	CSO	National	Advocacy, communication, social mobilisation
P11	F	Donor	National	Programme design and management
P12	F	IP	National	Programme management, systematic (active & passive) screening for active TB
P13	F	NTP/IP	National	Monitoring & evaluation, TB research

*CBO* Community based organisation, *CSO* Civil Society Organisation, *IP* Implementing Partner, *NTP* National Tuberculosis Programme

### First stakeholder engagement: Delphi 1

We developed the tool for Delphi 1 based on the literature [32]. CU conducted a scoping review, using systematic searches, snowballing from references of identified papers, and grey literature search to identify 17 systematic active and passive TB screening approaches with the potential to overcome gender-related barriers to TB care. The authors, CU, TW and JB, modified the list for coherence, resulting in 15 approaches. The Delphi tool listed the interventions, explained each with practical examples, and required/ asked participants to judge how useful each intervention could be in surmounting the male gender-related barriers to TB care in Nigeria and to substantiate with examples. Participants could also select a *'no response'* option. The Delphi tool was reviewed by the community advisory board for LIGHT Nigeria and piloted amongst two TB program managers who were not involved in the subsequent co-creation. CU emailed the Delphi tool (Supplementary material 1) to participants and followed up with weekly reminders. Between May and September of 2022, 13 out of 15 participants, who initially agreed to participate in the study, responded and clarifications about responses completed.

We applied a qualitative framework analysis approach [30] which offered a systematic way of identifying patterns within sets of qualitative data. CU indexed data, using NVivo software version 12 pro, and mapped responses using a spreadsheet, with interventions in columns and participants in rows. CU and BR tallied preferences and, together with TW, analysed for patterns of commonalities and synthesised summaries for each intervention.

### Second stakeholder engagement: Delphi 2

We designed a Google-based mobile-friendly tool with two key sections. The first section required stakeholders to rank the perceived likelihood of each intervention to successfully overcome gender-related barriers to TB care among men in Nigeria on a Likert scale (ranging from *‘lowest impact’* = *1, ‘low impact’* = *2, ‘moderate impact’* = *3, ‘high impact’* = *4, to 'highest impact'* = *5*) and to justify their ranking using optional free-text responses. The second section required participants to identify intervention packages by selecting (‘pick-and-drop’) up to three interventions expected to be feasible and acceptable, and perceived to have the highest combined effect in increasing male access to TB care. Participants were once again encouraged to provide justifications for their selections/choices through optional free-text responses. Before implementation, the tool was piloted as above. CU emailed the tool to all 13 participants and received all their responses within the month of October 2022.

Afterwards, the Likert scores were summed for each ranked intervention (Supplementary material 2) and were complemented by content analysis [31] of provided justifications where applicable. Inclusion decisions were made iteratively at post-analysis meetings. We considered interventions that were most frequently included in proposed packages, scored highest in the ranking, and/or gained strong positive justifications from the participants for inclusion in packages to be discussed at the participatory workshop.

### Third stakeholder engagement: Participatory workshop

**Fig. 1:**
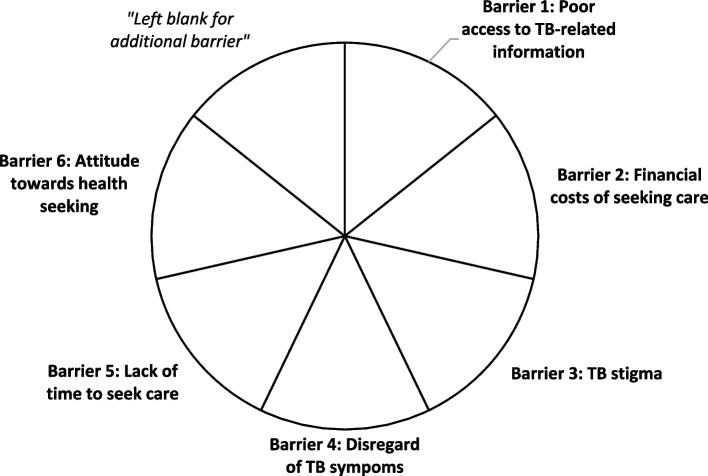
Intervention package analysis chart used at the workshop. Note. Each segment of the chart represents one of six barriers identified from the preceding qualitative research, with an additional segment for groups to include any other/ further barrier. Groups coloured the segments in accordance with their perceived extent to which the assessed intervention package could effectively address these barriers

All 13 participants were invited to take part in an in-person co-creation workshop aimed at achieving consensus on the most viable intervention. The one-day workshop took place in November 2022 and lasted for about six hours including a short break. CU introduced the intervention packages selected in the previous phase to TB stakeholders and assigned them into three separate groups. To facilitate the assessment of these packages, a visual tool (Fig. 1) was provided. Each group discussed and agreed on how well the assigned intervention package could address key barriers to TB care faced by men through shading corresponding segments in the circular chart provided. Afterwards, all three groups reconvened in a plenary session for discussions followed by foot voting and further discussions by stakeholders to reach consensus (Supplementary material 3).

Findings presented in this article are based on our review and synthesis of data from Delphi 1 and 2 and the workshop (flipcharts, notes, and video recording). CU and BR independently reviewed data, identified patterns underpinning decision-making along the consensus building process, and through iterative group discussions with TW, recognised three overarching themes. Overall, a combination of framework thematic analysis, descriptive statistical tallying, and content analysis were used to achieve the final themes.

### Quality assurance

The consensus building approach described in this manuscript carries risk of bias, including selection bias [25] and a tendency for researchers to lead the panel towards pre-determined ends [33]. To mitigate against such risk, participants were recruited using set criteria and researchers did not present them with specific data about any intervention to be assessed. Researchers held regular reflection sessions and made decisions collectively following discussion. The nature of engagement with stakeholders grew from being consultative to joint decision-making and power-sharing during the workshop [34]. CU, as lead researcher, remained critically aware of this positionality.

Power dynamics amongst participants can undermine the process and success of co-creation approaches [35, 36]. We anticipated the potential for the power asymmetry between participants to negatively affect participation in the process as our study included people of varying educational and professional levels and categories. To minimise this risk, we selected participants with relatively similar levels of experience in the TB field (minimum 10 years’ experience in the field) which helped to narrow gaps in TB-related knowledge and programme implementation experience. We also selected well established TB advocates recognised locally by the TB programme. While facilitating the in-person workshop, CU encouraged mutual respect for all stakeholders to participate in the decision-making of each group, whereas JB, OA, and RT observed and supported groups to facilitate equal participation.

## Results

This triphasic consensus building process mobilised knowledge from the experiences of multidisciplinary and multisectoral Nigerian stakeholders who scrutinised 15 possible interventions, suggested combinations of interventions, and agreed an intervention package deemed suitable for overcoming gendered barriers to TB care. Three themes summarise the outcomes of the consensus building: (1) preference for interventions proactively reaching men, (2) increasing awareness essential to systematic screening for TB, and (3) package of complementary and feasible TB interventions.

### Preference for interventions proactively reaching men

Table 3 summarises stakeholders’ views on the interventions, weighing perceived benefits and drawbacks of each approach. Overall, stakeholders prioritised active over passive approaches to screening. Interventions with a potential to quickly generate demand for services (like awareness creation and incentives) or increase yield (like chest x-ray screening) were also preferred over longer-term interventions (like policy solutions, healthcare worker training and health insurance). Generally, stakeholders at managerial levels raised concerns around the sustainability of interventions that require significant and continuous funding whilst acknowledging their potential usefulness in the long-term.

**Table 3:** Stakeholders’ intervention preference

Intervention (number of responses)	Overall feedback	Advantages reported	Disadvantages reported
**Interventions selected for Delphi 2**			
TB Screening in men’s socio-cultural congregate settings (*n* = 13)	Mostly positive views, selected for next stage for perceived acceptability, feasibility, and impact among men	• Reaches high numbers of men • Increases access to TB screening and information for men • Feasible and acceptable	-
Targeted awareness creation (*n* = 13)	Mostly positive views, selected for next stage for perceived effectiveness and complementarity	• Highly complementary to all approaches • Effective approach in many states • Creates/ generates demand for services through improved TB knowledge and awareness • Community-driven awareness campaigns work best	-
House-to-house screening (*n* = 13)	Mostly positive views, selected for next stage for its strong linkage to care	• Supports linkage to TB testing and care • Helps with treatment support, follow-up, and preventive therapy	• “Usual” timing more favourable for women than men; male-specific timing possible • Cost and time intensive
Workplace-based TB screening (*n* = 12)	Mostly positive views, selected for next stage for perceived high acceptability and impact among men	• Reduces men’s cost and time for hospital visit • Perceived high acceptability by men • Focus on high-risk workplaces (e.g., mines)	• May be cost intensive • Lack of operational guidance,
Family-based TB screening (*n* = 13)	Mixed views, selected for next stage for perceived effectiveness and potential to mitigate against TB stigma	• Increases reach to families and extended families • Promotes confidentiality with minimal stigma	• Challenges to reach male family heads • Cost, labour, and time intensive
Use of Chest X-ray screening (digital or non-digital) (*n* = 13)	Mixed views, selected for next stage for perceived effectiveness and advantages to the diagnosis of TB in men	• Useful for people with TB who are not exhibiting typical symptoms • Improves effectiveness of TB screening • Diagnose TB in men who deny or ignore symptoms	• Expensive equipment required • Special skillset required
Market-based case finding *(interventions in local open-markets)* (*n* = 12)	Mixed views, selected for next stage for perceived potential effectiveness in increasing TB diagnosis in men in markets frequented by men	• Established approach for health education • May improve access to TB screening and diagnosis • Types of markets are gendered, determining reach of men or women	• Associated with high pre-treatment loss to follow up
Incentives (conditional or unconditional cash transfers or non-cash/in-kind transfers) (*n* = 12)	Mixed views, selected for next stage for perceived effectiveness in reaching men and social protection of TB-affected families	• Alleviates TB-related catastrophic costs for households and improves TB outcomes • Increases uptake of non-TB-related health services	• Challenges of sustainability • Incentive may increase risk of fraud (data falsification)
Community TB outreaches and chest camps (*n* = 13)	Mixed views, selected for next stage for perceived high impact	• Established, impactful systematic active screening approach in the TB program	• Usually, not cost-effective • High tendency to reach more women and children • Dependent on proper planning and mapping
**Interventions not selected for Delphi 2**			
Peer-led intervention *(interventions led and implemented entirely by peers. Could be men, women, or young people)* (*n* = 7)	Mostly negative views, deselected for perceived time implications and perceived risk of stigmatisation of peer educators	• Popular amongst women in some areas	• Requires much training for the peer leaders • Not acceptable; high reluctance because of TB-related stigma
Health insurance schemes, disability and sickness allowance (*n* = 9)	Mostly negative views; deselected for absence of functional health insurance schemes in many sub-national settings	• May reduce TB catastrophic cost for families • Institutionalises sustainable support for TB services • Can reduce delay and inertia to care seeking	• Lack of functional health insurance schemes in many states in Nigeria
Flexible clinic hours (*n* = 11)	Mostly negative views, deselected for perceived risk of overburdening healthcare teams and uncertainty of effectiveness	• Useful approach for people who work	• Limited in that it relies on a passive approach to screening for active TB • Not feasible in facilities with few healthcare workers or staff shortages
Policy intervention(s) (*n* = 9)	Mostly negative views, deselected for uncertainty of effectiveness and time implications (inability to implement within a short time)	• May address broader social determinants	• Effective in the long run, not in the short-term • Conditional on stringent implementation and enforcement • Subject to political will and funding
Training of Healthcare workers in gender-responsive approaches to care (*n* = 10)	Mostly negative views, deselected for perceived uncertainty of effectiveness and cost implications	• May make delivery of TB services more gender-responsive • May make TB services more person-centred	• Training alone ineffective to increase access for men • Can be resource-intensive
Female-led interventions *(interventions led and implemented entirely by females e.g. Uwar’ gida)* (*n* = 8)	Mostly negative views, deselected for lack of confidence in ability to reach men	-	• Popular and useful in enhancing women’s access to reproductive, maternal, child health services, but may not improve access to health services for men • May not reach men due to culture

Proactive screening and testing in communities, families, homes, markets, workplaces, social and cultural places where men congregate were ranked as having high potential to reach men. House-to-house—also called “door-to-door”—TB screening was valued for easing linkage to care and treatment support among people diagnosed with TB.

*“there is minimal risk of having loss-to-follow up as patients are tracked/identified from their places of residence” (P8)*.

Nonetheless, stakeholders raised concerns about the timing of community-based and house-to-house TB screening, unlikely to help reach men, and about associated costs.

*“This approach is one of the community active TB case-finding that is more likely to improve access to women than men if implemented in the conventional way (mornings through afternoons). However, if the implementation approach includes after working hours, it can also increase access to TB services for men too.” (* ***P1*** *).*

*“Many community-based TB case finding approaches incorporate house-to-house TB screening and active case finding. However, although some TB cases have been found using this approach, it is not usually cost effective” (* ***P2*** *).*

Views about interventions in local markets were mixed with it being considered more useful for TB information than for screening. Whilst many people can be found at the market, engaging them during their shopping and linking them to TB testing and care afterwards was viewed as challenging.

*“From my experience, market-based case finding has worked more for awareness creation than case finding as most persons are focused on the purpose of market visit. It is therefore associated with limited case finding.” (* ***P10*** *).*

*“This [market-based approach] gives the opportunity to have access to a huge number of people for screening and testing. It is also an opportunity to create awareness to many people at the same time. The drawback is the probability of having pre-treatment loss to follow-up among identified cases [sic]” (* ***P9*** *).*

Most of the interventions relying on passive approaches to finding people with active TB were deprioritised. Stakeholders reported that peer- and female-led interventions required a lot of training and support whilst not aiding in men’s access to TB services due to the perception, especially among women, of difficulties in convincing men to seek care. They also viewed changes of enhancing TB diagnosis among men through training of healthcare workers or the extension of clinic hours as unfeasible and/or unproductive on their own.

*“This [extension of clinic hours] approach can increase access for working class men and businessmen who may not be able to attend the clinics due to conventional clinic working hours/days. However, this approach may only give access to those who are truly willing to come to clinics. There are those who, unless they are seriously ill, may still not attend clinics. To me, this approach is more a passive case finding approach than active… In health facilities where there are only few staff, especially in some remote communities, this approach might not be feasible”. (****P7****)*

### Awareness creation essential to systematic screening for TB

**Fig. 2:**
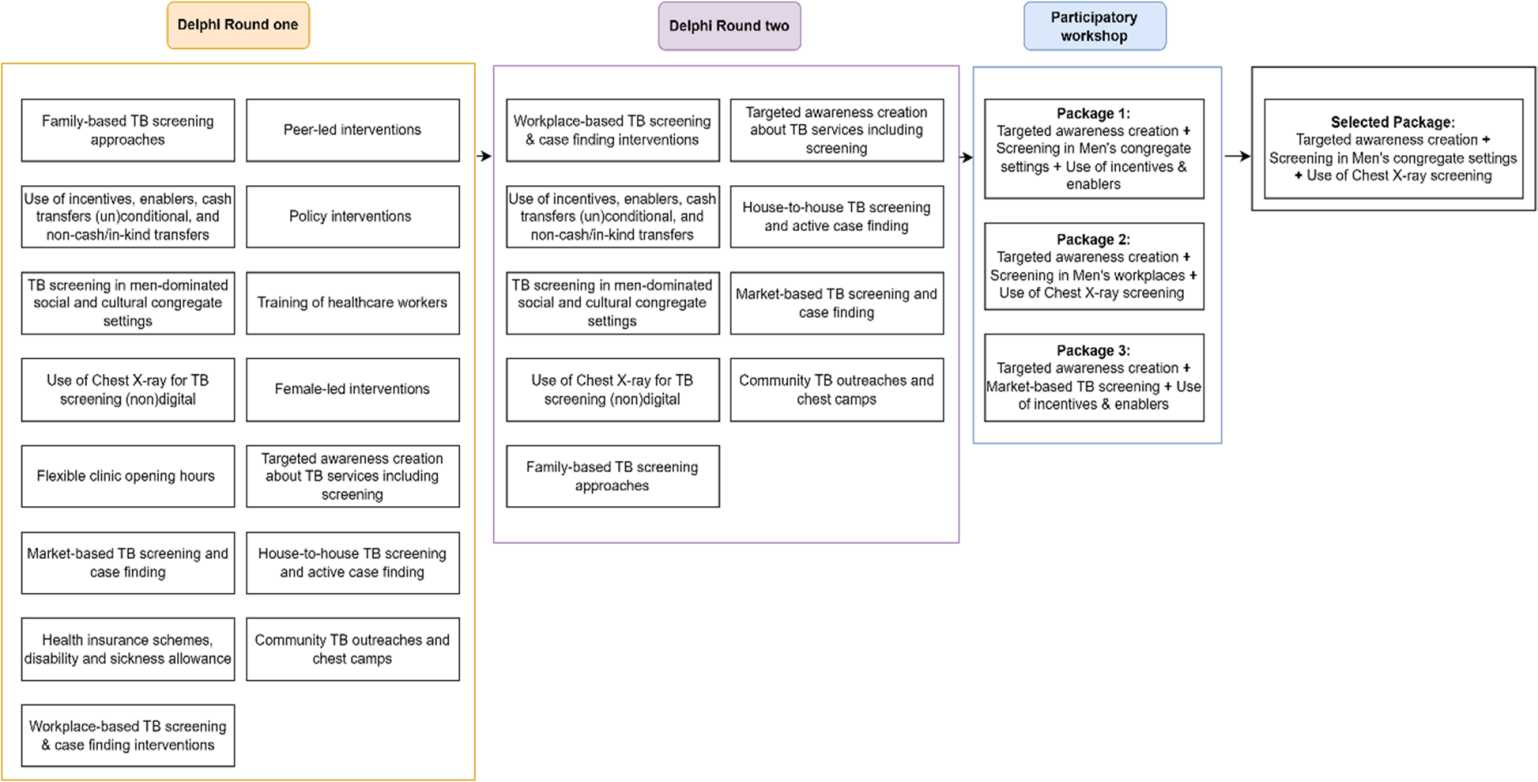
Progression of interventions through the stages of collaborative stakeholder engagement

Alongside six variations of community-based screening strategies, additional approaches of awareness creation, incentives, and chest X-ray aided screening were considered for inclusion in service packages in the second stage of the study (Fig. 2). Details of the scoring and packaging are included in the supplementary file 2.

Overall, there was consistency in the importance of targeted awareness creation to amplify the gains from any chosen intervention by increasing uptake. Stakeholders included targeted awareness creation most frequently (highest tally = 20, third highest Likert score = 47/65) as an important component of any chosen intervention package.

*“This targeted awareness creation could greatly improve access to TB services in a gender-sensitive manner. This might be… reaching women groups in markets or men in bars (beer parlours) and drinking arena in community settings. Targeted awareness creation will include reaching groups in the community…. An example is a targeted awareness creation among bus drivers or tricycle operators in major cities of Nigeria. Others are social groups of hair stylists, barbers, etc. Targeted awareness campaigns focused on such groups could improve access to TB care.” (* ***P10*** *)*

*“Targeted awareness creation has been done as part of approaches that combine active TB case finding of men in congregate settings towards improving TB case finding. In my experience, targeting male social or religious gathering has been successful in improving TB knowledge/awareness creation, demand creation for TB services and in driving key lessons regarding TB case finding to men. This approach was utilised in Zamfara and Sokoto States to improve TB case finding in their States”* ***(P9)***

Three variations of active screening for TB among men ranked highly and thus were included in proposed packages: Screening in men’s socio-cultural congregate settings (tally = 14, highest Likert score = 52/65) reflecting stakeholders’ high expectations for reaching men; whilst market and workplace-based approaches tallied enough to merit inclusion in final stage, their relatively lower Likert scores (36/65 and 40/65, respectively) reflect stakeholders’ concerns regarding effectiveness. Although they could enhance access to TB screening for men and women, high levels of pre-treatment loss to follow up remained major concerns, especially around market-based interventions. Conversely, house-to-house TB screening, usually associated with minimal losses to follow up, scored highly (tally = 8) but was perceived to be greatly inhibited by high implementation cost and manpower. Stakeholders posited that *“House-to-house depends on the time of visit. You will meet more females during certain time of the day than males and children” (P3).* It also needs to be well targeted at high burden settings with home visitors needing to work late into the evenings in order to reach men. Incentives, enablers, cash-transfers and non-cash commodities were also favoured (tally = 14, Likert score = 45/65), despite stakeholders raising issues regarding the challenges of sustaining such interventions. Stakeholders stressed direct protective effect of economic support to mitigate catastrophic costs as *“many families were living below the poverty line”* (**P9)**.

*“Incentives are pivotal especially at the early stages of case-finding when the numbers are minimal. Current data shows that 71% of TB patients are exposed to catastrophic spending to meet their care needs in Nigeria. […]*

*Such incentives/enablers are a critical part of social safety nets which are near to non-existent in Nigeria. Social safety nets are important in improving control and response to infectious disease such as TB.”(P11)*

Chest X-ray aided TB screening was deemed to help overcome men’s dismissive attitude toward early TB symptoms and enable early diagnosis (tally = 14, Likert score of 46/65). It was considered.

*“…one of the very high impact interventions as it helps to identify presumptive in seemingly normal persons and increases the number of clinically diagnosed TB which has remained a big challenge to TB notification in Nigeria” ****(P10)***.

### Package of complementary and feasible TB interventions

The stakeholder workshop revealed that feasibility and considerations of the complementarity of interventions were crucial to the final decision on inclusion of interventions into the preferred package as well as their predicted effectiveness to address the key barriers. Targeted awareness creation, which was part of all three packages, was perceived as an essential component to increase knowledge, uptake of interventions, and support men to make informed health choices related to TB. Participants reported that men in the communities have limited TB-related knowledge/awareness including the seriousness of the symptoms, transmission risk within their households, where to find care in their communities, and the fact that TB tests and anti-TB medicines were provided free of charge. Additionally, men’s attitude towards seeking healthcare and feelings of stigma were also identified as areas that could be addressed by components of the awareness messaging. TB screening in markets, workplaces, and socio-cultural male congregate settings all sought to proactively bring the service entry point closer to the men, thereby reducing the cost and time of seeking care, whilst the use of incentives aims to protect people against adverse economic outcomes of TB.

Voting did not lead to a clear preference for any single intervention package. Package 1 (awareness creation, screening in men’s congregate settings, and incentives) gained greatest support (*n* = 7), followed by a modification of package 1 (awareness creation, screening in men’s congregate settings, and X-ray screening) (*n* = 4) and package 2 (awareness creation and workplace-based screening using chest x-ray) (*n* = 2). Although Package 1 was voted more times, its programmatic sustainability was discussed and the use of incentives was not considered suitable. A participant who voted for package 2 strongly felt that *“chest X-ray screening has been a game changer for the TB program”* since portable digital X-ray technologies have become more widely available in the Nigerian TB landscape. This argument convinced others and participants agreed an entirely new package comprising ‘targeted awareness creation, TB screening in men’s congregate settings, and use of chest x-ray screening”.

## Discussion

In our approach, TB stakeholders supported the selection of a viable gender-responsive TB intervention package for men to be evaluated as part of the DESTINE study in peri-urban settlements of Nigeria. Our method, a departure from the usual top-down approach of developing interventions common in the TB control landscape [37], was well-received by TB stakeholders. Starting with a long list of potential interventions, TB stakeholders achieved consensus through two iterations of the Delphi process and a participatory workshop. Delphi required stakeholders to make individual choices based on their experiences, whilst the participatory workshop afforded them the time to fine-tune their thoughts during in-person interactions across disciplines and sectors. Stakeholders’ responses suggest a preference for interventions designed to proactively reach men, while emphasising the importance of awareness creation as a fundamental component to any form of systematic screening for TB, as well as agreeing on a package that accounts for complementary and feasible TB interventions. In summary, given the absence of best practices for gender-responsive TB interventions in our context, leveraging experiences of Nigerian stakeholders through iterative engagements yielded consensus on a package of TB interventions that could potentially address barriers faced by men in reaching care for TB.

Stakeholders concluded that combining awareness creation and screening in men’s congregate settings using (digital) chest X-ray (Fig. 2) has the greatest potential to improve access to TB care for men. A nationwide survey commissioned by the National Tuberculosis Leprosy and Buruli Ulcer Control Programme found low levels (27%) of accurate TB knowledge in communities [38]. Awareness creation, on its own, has been shown to be insufficient to induce behaviour change [39]. However, in combination with other systematic screening approaches, awareness creation can increase the detection of TB cases among both men and women [40]. The stakeholders posit that challenges such as poor/ inadequate access to information, stigma, and masculine health-seeking behaviour will be addressed by a ‘men-led targeted TB and health awareness creation’. The health-seeking behaviour messaging will not only focus on TB but will aim to challenge and potentially transform the masculine gender norm that discourages men’s engagement in the process of healthcare generally [41]. Bringing the entry point of TB services closer to men in their communities potentially eliminates financial costs and minimises the time involved in seeking care. Chest X-ray mediated TB screening has a long history of implementation across the world [42, 43] and in Nigeria [44] with significant impact on identification of people with the disease. A nationwide systematic screening of over 21 million people found that interventions that included x-ray mediated screening led to greater yield compared to those using only the WHO four symptom screening [11]. Its use for screening has the potential to counteract the tendency for men to dismiss TB symptoms such as persistent cough, usually considered insufficient to warrant a hospital visit, and thus will lead to earlier diagnosis of TB. Thus, the DESTINE intervention package is a novel combination of interventions that have documented effectiveness to improve TB treatment outcomes. Although the intervention was designed to reach the largest proportion of men at risk of TB in these peri-urban communities, it was also purposefully designed to be adaptable to their heterogenous needs. The TB-related awareness creation led by men – and indeed mobile x-ray screening outreach—can be conducted in diverse settings including football fields and viewing centres (to reach adolescent boys and young men); mines (to reach mine workers) and other settings in which vulnerable men of any age or socio-economic group may congregate.

The research outcome was dependent on stakeholders’ views and experiences expressed at all stages of the process. We acknowledge the limitations due to the study sample. We could only involve stakeholders with functional email addresses and sufficient literacy to provide written responses to Delphi. We engaged experienced TB stakeholders and might have missed unconventional views from stakeholders who are less experienced. TB stakeholders from community or local government level, who have been instrumental in the design of systematic TB screening interventions elsewhere [45], were underrepresented in the sample. We recognise the importance of policy and programmes that address the intersectionality of gender and social determinants [46, 47] but that was beyond the scope of the current research. Despite these limitations, our study offers a methodology for collaborative intervention prioritisation and selection processes and a gender-responsive TB intervention package for men in peri-urban settlements for advancing person-centred TB care in Nigeria.

## Conclusion

Leveraging experiences of TB stakeholders through iterative engagements has led to a consensus on a gender-responsive TB intervention package, combining awareness creation with systematic active screening and novel technology (CXR) to reach men with TB in Nigeria. Clear priority was given to approaches proactively seeking to increase TB knowledge and identification of men with TB symptoms. The intervention package will be implemented collaboratively with communities and CBOs as part of the DESTINE study and will be evaluated for feasibility, acceptability, and cost. Men from peri-urban settlements will be involved in selecting men’s sociocultural congregate places for systematic screening and key messages for awareness creation to mitigate against the risk of further alienating men as *reservoirs* of illness in the community. Future research is needed to adapt and test our consensus-building model for priority setting in diverse fields and contexts; as well as evaluating the effectiveness of the gender-responsive TB intervention package for men in peri-urban settings.

## Supplementary Information

Supplementary Material 1.

Supplementary Material 2.

Supplementary Material 3.

## Data Availability

No datasets were generated or analysed during the current study.

## Abbreviations

CBOs: Community based organisations
CSO: Civil society organisation
CXR: Chest X-ray
DESTINE: Development and Evaluation of gender-reSponsive TB Intervention in NigEria
LIGHT: Leaving no one behind: Transforming gendered pathways to health for TB
NTP: National Tuberculosis programme
IP: Implementing partner
