# Supplementary material for: Co-creation of a gender responsive TB intervention in Nigeria: a researcher-led collaborative study
Source: BMC Health Serv Res. 2025 Jan 13;25:63. doi: 10.1186/s12913-025-12241-7 (PMC11726931; doi:10.1186/s12913-025-12241-7)
Supplement: Supplementary file 1 — Supplementary Material 1. [file 12913_2025_12241_MOESM1_ESM.docx]

**COREQ (COnsolidated criteria for REporting Qualitative research)**

**Co-creation of a gender responsive TB intervention in Nigeria: a researcher-led collaborative study**

**Chukwuebuka Ugwu et al.**

| Topic Guide | Item No. | Questions/Description | Reported on Page No. |
| --- | --- | --- | --- |
| **Domain 1: Research team and reflexivity** |  |  |  |
| *Personal characteristics* |  |  |  |
| Interviewer/facilitator | 1 | Which author/s conducted the interview or focus group? | CU |
| Credentials | 2 | What were the researcher’s credentials? E.g. PhD, MD | MBBS  MPH  PhD (Thesis writing) |
| Occupation | 3 | What was their occupation at the time of the study? | Early Career Researcher and full time PhD student |
| Gender | 4 | Was the researcher male or female? | Male |
| Experience and training | 5 | What experience or training did the researcher have? | Advanced training in qualitative research methods. Safeguarding, Ethics, and Good clinical practice. |
| *Relationship with participants* |  |  |  |
| Relationship established | 6 | Was a relationship established prior to study commencement? | Yes. |
| Participant knowledge of the interviewer | 7 | What did the participants know about the researcher? e.g. personal goals, reasons for doing the research | Yes. The researcher explained the research goal and emailed copies of the Participants’ information leaflet to each participant. |
| Interviewer characteristics | 8 | What characteristics were reported about the interviewer/facilitator? e.g. Bias, assumptions, reasons and interests in the research topic | A discussion of the positionality of the researchers is included in the manuscript. |
| **Domain 2: Study design** |  |  |  |
| *Theoretical framework* |  |  |  |
| Methodological orientation and Theory | 9 | What methodological orientation was stated to underpin the study? e.g. grounded theory, discourse analysis, ethnography, phenomenology, content analysis | This research utilised consensus building approach and applied content analysis methods. |
| *Participant selection* |  |  |  |
| Sampling | 10 | How were participants selected? e.g. purposive, convenience, consecutive, snowball | The key informants that were engaged for this consensus building were selected purposively. |
| Method of approach | 11 | How were participants approached? e.g. face-to-face, telephone, mail, email | The participants were first called by CU explaining the overview of the research. Thereafter, a detailed email containing further guidance, and the PIS was sent to each person. |
| Sample size | 12 | How many participants were in the study? | 15 participants agreed to take part in the study. |
| Non-participation | 13 | How many people refused to participate or dropped out? Reasons? | Two did not respond to reminder emails and calls and gave no reason for not continuing. |
| *Setting* |  |  |  |
| Setting of data collection | 14 | Where was the data collected? e.g. home, clinic, workplace | The first two stages of the data collection were written responses transmitted per email to CU while the third stage was a one-day in-person workshop. |
| Presence of non-participants | 15 | Was anyone else present besides the participants and researchers? | Yes, the trained media officers were present. |
| Description of sample | 16 | What are the important characteristics of the sample? e.g. demographic data, date | Details of the participants were captured in Table 2. |
| *Data collection* |  |  |  |
| Interview guide | 17 | Were questions, prompts, guides provided by the authors? Was it pilot tested? | Yes. The questions were piloted and were emailed to the participants. |
| Repeat interviews | 18 | Were repeat interviews carried out? If yes, how many? | NA |
| Audio/visual recording | 19 | Did the research use audio or visual recording to collect the data? | All Delphi interviews were received as written text already and the video recording made was done with intent of record keeping rather than data collection. |
| Field notes | 20 | Were field notes made during and/or after the interview or focus group? | Yes, field notes were taken during the in-person workshops. |
| Duration | 21 | What was the duration of the interviews or focus group? | The in-person workshop lasted 6 hours with an hour break for refreshments in between. |
| Data saturation | 22 | Was data saturation discussed? | NA. |
| Transcripts returned | 23 | Were transcripts returned to participants for comment and/or correction? | Not Applicable as all Delphi data came in text form. |
| **Domain 3: analysis and findings** |  |  |  |
| *Data analysis* |  |  |  |
| Number of data coders | 24 | How many data coders coded the data? | Three. CU, BR, and TW. |
| Description of the coding tree | 25 | Did authors provide a description of the coding tree? | NA. |
| Derivation of themes | 26 | Were themes identified in advance or derived from the data? | No. |
| Software | 27 | What software, if applicable, was used to manage the data? | NVivo and MS Excel. |
| Participant checking | 28 | Did participants provide feedback on the findings? | At the in-person workshop, the participants produced the findings. |
| *Reporting* |  |  |  |
| Quotations presented | 29 | Were participant quotations presented to illustrate the themes/findings? Was each quotation identified? e.g. participant number | Yes. |
| Data and findings consistent | 30 | Was there consistency between the data presented and the findings? | Yes. |
| Clarity of major themes | 31 | Were major themes clearly presented in the findings? | Yes. |
| Clarity of minor themes | 32 | Is there a description of diverse cases or discussion of minor themes? | Yes. |

Source: Tong, A., Sainsbury, P., & Craig, J. (2007). Consolidated criteria for reporting qualitative research (COREQ): a 32-item checklist for interviews and focus groups. *International Journal for Quality in Health Care*, 19(6), 349-357. doi:10.1093/intqhc/mzm042
