# Supplementary material for: Co-creation of a gender responsive TB intervention in Nigeria: a researcher-led collaborative study
Source: BMC Health Serv Res. 2025 Jan 13;25:63. doi: 10.1186/s12913-025-12241-7 (PMC11726931; doi:10.1186/s12913-025-12241-7)
Supplement: Supplementary file 2 — Supplementary Material 2. [file 12913_2025_12241_MOESM2_ESM.docx]

| Full document number: | N/A |
| --- | --- |
| Document name: | DESTINE Delphi Guide |
| Version: | 4.0 |
| Superseded version: | 3.0 |
| Originator job title: | PhD/ECR |
| Reason | Comments on key improvements by supervisor |
| Add document to LSTM website: | N/A |
| Authorship date: | 28/06/2022 |
| Approved by: | Supervisor: Dr. Tom Wingfield |
| Approval date: |  |
| Next review date: | N/A |

Development and evaluation of gender-sensitive TB interventions for community settings in Nigeria.

Delphi Interview Guide.

| **Concept** | ***Question:***  *From your experience with people with TB and/or the National TB Program and/or wider knowledge please describe how each of these approaches can or could practically help to overcome gendered barriers faced by men and women in accessing TB care?*   - *Feel free to use examples from Nigeria, Africa and beyond where relevant* - *Tick ‘no knowledge/experience’ where applicable (we will follow up if a box is left entirely blank)* |
| --- | --- |
| **Workplace-based TB screening and case finding interventions**  *Active case finding in conducted in places of work dominated by men (or women)* | No Knowledge/experience |
| **Family-based TB screening and case finding approaches**  *(Interventions that target the family unit using one member of the family as entry point)* | No Knowledge/experience |
| **Use of Incentives, enablers, cash transfers (conditional or unconditional), and non-cash/in-kind transfers**  *(Use of various forms of incentives or enablers to encourage care seeking* *Includes things like non-cash transfers such as food / travel vouchers / insurance etc.)* | No Knowledge/experience |
| **Men’s congregate settings**  *(TB screening and active Case finding in places where men congregate socially, culturally or religiously such as mosques, churches, majalisa, bars etc.)* | No Knowledge/experience |
| **Chest X-ray screening (digital or non-digital)** | No Knowledge/experience |
| **Flexible clinic hours**  *(e.g.: weekends or evenings)* | No Knowledge/experience |
| **Market-based case finding**  *(interventions in local open markets)* | No Knowledge/experience |
| **Health insurance schemes, disability and sickness allowance** | No Knowledge/experience |
| **House-to-house TB screening and active case finding** | No Knowledge/experience |
| **Community TB outreaches and chest camps**  *(Including mobile clinics, WOW trucks etc.)* | No Knowledge/experience |
| **Peer-led intervention**  *(Interventions designed and implemented by men or women among their peers)* | No Knowledge/experience |
| **Policy intervention(s)**  *Enactment of policies that will help overcome some of the gendered barriers such as stigma, work benefits etc.* | No Knowledge/experience |
| **Training of Healthcare workers**  *(Training on gender-sensitive care giving)* | No Knowledge/experience |
| **Female-led interventions**  *An example is the house-wife (Uwar-gida) approach* | No Knowledge/experience |
| **Targeted awareness creation**  *(ACF that includes intensifying awareness amongst a group or community as a whole)* | No Knowledge/experience |
| **Other** (please specify)  *(If you have knowledge or intervention not listed above boxes, please specify here)* | No Knowledge/experience |
