# Supplementary material for: Co-creation of a gender responsive TB intervention in Nigeria: a researcher-led collaborative study
Source: BMC Health Serv Res. 2025 Jan 13;25:63. doi: 10.1186/s12913-025-12241-7 (PMC11726931; doi:10.1186/s12913-025-12241-7)
Supplement: Supplementary file 3 — Supplementary Material 3. [file 12913_2025_12241_MOESM3_ESM.docx]

Supplementary material 2 – **Intervention Scoring sheet and packages tally sheet**

| **Table showing stakeholder Likert scores for interventions during the second round of Delphi survey**  **Instructions**: The new shortlist of potential gender-sensitive TB case finding interventions developed following Stage 1 of the Delphi is shown below. Kindly rate how impactful you consider each single intervention would be at successfully finding more men (and women) with TB. | | | | | | | | |
| --- | --- | --- | --- | --- | --- | --- | --- | --- |
| **Workplace-based TB screening** | **Targeted TB screening awareness creation** | **TB Screening in men's socio-cultural congregate settings** | **Market-based TB screening** | **Use of Incentives, Enablers, cash-transfers and non-cash** | **House-to-House TB screening** | **Family based TB interventions** | **Community outreaches** | **Chest X-ray screening** |
| 3 | 3 | 5 | 3 | 3 | 3 | 3 | 3 | 1 |
| 2 | 3 | 4 | 4 | 4 | 4 | 3 | 3 | 5 |
| 5 | 5 | 5 | 4 | 3 | 3 | 3 | 5 | 4 |
| 4 | 5 | 5 | 4 | 5 | 5 | 5 | 5 | 4 |
| 2 | 4 | 2 | 3 | 3 | 5 | 2 | 5 | 4 |
| 3 | 4 | 5 | 3 | 3 | 3 | 4 | 3 | 5 |
| 1 | 3 | 4 | 3 | 5 | 2 | 3 | 3 | 3 |
| 3 | 5 | 5 | 4 | 4 | 5 | 5 | 4 | 4 |
| 3 | 3 | 4 | 4 | 5 | 3 | 3 | 4 | 4 |
| 3 | 4 | 5 | 3 | 5 | 5 | 4 | 4 | 4 |
| 4 | 5 | 5 | 3 | 3 | 4 | 5 | 5 | 5 |
| 3 | 3 | 3 | 2 | 2 | 4 | 4 | 4 | 3 |
| **36** | **47** | **52** | **40** | **45** | **46** | **44** | **48** | **46** |

| **Table showing the frequency of packaging for each intervention during the second round of Delphi survey**  **Instructions**: Please select two or more interventions that you consider could be combined/integrated into a package that would have greater impact than the constituent single interventions alone. | | | |  |
| --- | --- | --- | --- | --- |
|  | **Package 1** | **Package 2** | **Package 3** | **Total tally** |
| Workplace-based TB screening | 2 | 4 | 3 | 9 |
| Targeted awareness creation | 9 | 5 | 6 | 20 |
| Screening in Men's congregate settings | 7 | 4 | 3 | 14 |
| Market-based TB screening | 2 | 3 | 3 | 8 |
| Use of incentives Enablers Cash/Non-cash | 5 | 4 | 5 | 14 |
| House-to-House TB screening | 2 | 4 | 2 | 8 |
| Family-based TB intervention | 0 | 1 | 1 | 2 |
| Community Outreaches | 3 | 2 | 0 | 5 |
| Chest X-ray Screening | 4 | 4 | 3 | 11 |
